# Laboratory Characterization of Geosynthetics-Reinforced Asphalt Mixture

**DOI:** 10.3390/ma14216424

**Published:** 2021-10-26

**Authors:** Xianrong Wang, Xilong Zhou, Xuan Zhang

**Affiliations:** 1 School of Transportation and Logistics Engineering, Wuhan University of Technology, Wuhan 430000, China; wxr@whut.edu.cn; 2 Second Highway Company Co., Ltd., Wuhan 430000, China; 3 CCCC First Highway Engineering Group Co., Ltd., Beijing 100000, China; zhangxuan01@cfhec.com

**Keywords:** road engineering, geosynthetic-reinforced, dynamic stability, bending creep rate, fatigue life

## Abstract

In order to improve the mechanical properties of asphalt pavement, geosynthetics can be employed in asphalt mixture. This research designed 12 reinforced schemes based on the types of geosynthetics, bonding layers and reinforced position. For the relative tests carried out, reinforced specimens were prepared according to each individual scheme. Moreover, rutting tests, bending creep tests and split fatigue tests were carried out on reinforced specimens in the laboratory. The results obtained in this investigation showed that the dynamic stability, bending creep rate and fatigue life of geocell-reinforced specimens are better than those of geogrid-reinforced specimens. The bonding layer of Styrene-Butadiene-Styrene (SBS) modified asphalt is better than epoxy modified asphalt. The dynamic stability and fatigue life of middle reinforcement are better than those of the lower reinforcement, while the bending creep rate of the lower reinforcement is better than middle reinforcement. In addition, reinforced scheme (9) has the largest increase in dynamic stability and fatigue life by 103 and 137%, respectively, and reinforced scheme (12) has the largest reduction in bending creep rate by 46%. However, scheme (9) improved dynamic stability and fatigue life by 43 and 29% higher than scheme (12), while the reduction of flexural creep rate of scheme (12) is only 7% higher than that of scheme (9).

## 1. Introduction

Currently, asphalt pavements are subjected to high traffic volumes generating distresses such as rutting and cracking that need frequent and expensive maintenance [1,2]. In general, there are two fundamental approaches to soothe this issue: one is to frequently mill and apply a new asphalt overlay (mill and fill) at certain intervals of the pavement’s service life [3,4,5]. This approach is rather expensive due to a need for renewal over a short period of time, which in turn requires additional financial and material resources and does not correspond with the environmental concerns arising from releasing large amounts of emissions in the air along with high-energy consumption. Another option, which in recent years has drawn much attention from the engineering community, is to reinforce the bituminous pavement system by embedding geosynthetics in the asphalt layers [6,7,8,9,10,11]. In addition to the relatively considerable economic and social benefits, the second method also has a superior distress treatment effect.

As a common pavement distress, rutting will reduce the quality of pavement service and cause safety hazards. Bertuliene et al. [12] conducted a series of rutting tests to explain the role of geosynthetics in the asphalt layer, and the results showed that the rut depth of the reinforced specimens decreased by 40% compared with unreinforced. Correia et al. [13] used a self-made wheel loading device to cyclically load the geogrid-reinforced specimens, the results showed that compared with the unreinforced specimens, the rut depth of reinforced specimens was reduced by 40%, and the stress and strain at the bottom of the asphalt layer were reduced by 30 and 55%, respectively. Qadir et al. [14] used the multiple linear regression method to compare and analyze the anti-rutting performance of flexible and rigid geogrids when used to reinforce asphalt pavements. The test results showed that the application of geogrid as a reinforcement layer in asphalt pavement can improve the anti-rutting performance of the pavement, and the improvement of flexible geogrid reinforcement is higher than that of rigid geogrid reinforcement.

When the base layer produces shrinkage cracks or low-temperature shrinkage cracks, the contact position between the bottom of the asphalt layer and the crack is prone to stress concentration under the action of the vehicle, causing the asphalt surface to form a reflective crack. An experimental program was conducted to determine the effects of geosynthetic reinforcement on mitigating reflection cracking in asphalt overlays by Khodaii et al. [15]; results indicated a significant reduction in the rate of crack propagation in reinforced specimens compared to unreinforced specimens and geogrid position affected the type of crack propagation in asphalt overlays. Nejad et al. [16] studied the performance of geogrid and geotextile in asphalt overlay to delay the rate of reflective crack propagation, based on a regression model it was identified that the glass grid has the best effect on improving the overlay performance. Li et al. [17] used two hydraulic servo systems and an environmental box to make a joint motion simulation system, and used this device to study the process of reflection cracks in geotextile-reinforced concrete. The research results showed that reflective cracks expand from bottom to top, the length of the crack does not increase linearly with time, and its growth mode is in a stepped manner.

In addition to rutting and reflection cracks, fatigue cracks are also one of the main manifestations of asphalt pavement distress. Dawei et al. [18] applied geotextiles and geogrids to the asphalt layer, and compared their effects on the fatigue performance of asphalt mixtures. The results showed that the fatigue resistance of asphalt mixture is not significantly enhanced by geotextiles, only increased by 0.4%, while the geogrid increases the fatigue life of asphalt mixture by 20.6%. Arsenie et al. [19] presented a complete experimental study of the fatigue behavior of a glass-fiber-reinforced asphalt mixture, and the comparison of the fatigue curves of non-reinforced and reinforced asphalt mixture indicated that the geogrid can inhibit the expansion of fatigue cracks and increase the fatigue life by up to 50%; moreover, it is considered that the two most important factors affecting the fatigue life of a reinforced specimen are the test temperature and reinforced location. Kumar et al. [20] used digital image processing technology (DIC) to analyze the fatigue behavior of the geogrid and geotextile reinforced asphalt mixture beam specimens in the bending state. The results showed that the geotextile has little effect on the fatigue life of the specimen, and the geogrid can greatly increase the fatigue life of the asphalt concrete beam specimen.

Although the use of geosynthetics for reinforcement of asphalt pavement has been verified by most researchers in various aspects [21,22,23], the evaluation of the reinforced effect varies with the research content. For example, the improvement of crack resistance of geosynthetics is often ignored when analyzing its inhibition of reflection crack, and its effect on rutting resistance is also ignored when analyzing its anti-crack performance [24,25]. This makes the actual reinforcement effect and design analysis results always have a certain difference.

Given this background, the research presented in this paper aims to evaluate the effectiveness of asphalt mixture reinforcement with different reinforced schemes. To this purpose, rutting tests, bending creep tests and split fatigue tests were carried out on reinforced specimens in the laboratory. Two reinforcing materials, three bonding layers and two reinforced positions were considered for the realization of the laboratory specimens. Unreinforced reference systems were also included for comparison purposes.

## 2. Materials

### 2.1. Asphalt Concrete and Bitumen

The aggregate used for the specimen was basalt (Hubei Huanghuang highway management Co. Ltd., Wuhan, China), and the binder was SBS-modified asphalt (Hubei Huanghuang highway management Co. Ltd., Wuhan, China) which has been widely used in China. Considering the smaller grid size of geosynthetics in laboratory tests, the dense-graded AC-13 with a smaller particle size was selected for the gradation, as shown in Table 1. According to the Marshall test, the optimal asphalt content is 4.45%.

During the experimental investigation, two different types of grid coatings were applied at the interface of the geosynthetics: an SBS polymer modified asphalt and an epoxy modified asphalt (Hubei Huanghuang highway management Co. Ltd., Wuhan, China). Among them, epoxy modified asphalt is an irreversible cured product formed by adding epoxy resin to asphalt, and hardening reaction with curing agent. The main technical indicators are shown in Table 2. In both cases, 0.15 kg/m^2^ of residual bitumen was spread at the interface.

### 2.2. Reinforcing Material

**Figure 1:**
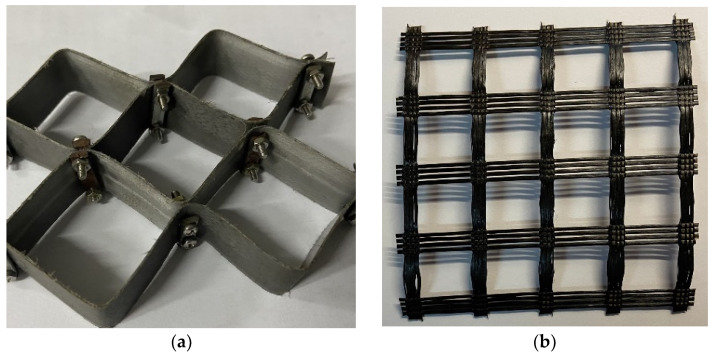
Reinforcing materials: (**a**) the PP geocell; (**b**) the GF geogrid.

The geocell used for this study consists in a Polypropylene (PP, Hubei Huanghuang highway management Co. Ltd., Wuhan, China) material that is known for its high strength and temperature resistance. As for the other reinforcing material geogrid, it is made of high-strength glass fiber (GF, Hubei Huanghuang highway management Co. Ltd., Wuhan, China) through the international advanced warp knitting process. The properties of PP geocell and GF geogrid (Figure 1) given by the manufacturer are shown in Table 3.

## 3. Laboratory Test

### 3.1. Reinforced Schemes

**Figure 2:**
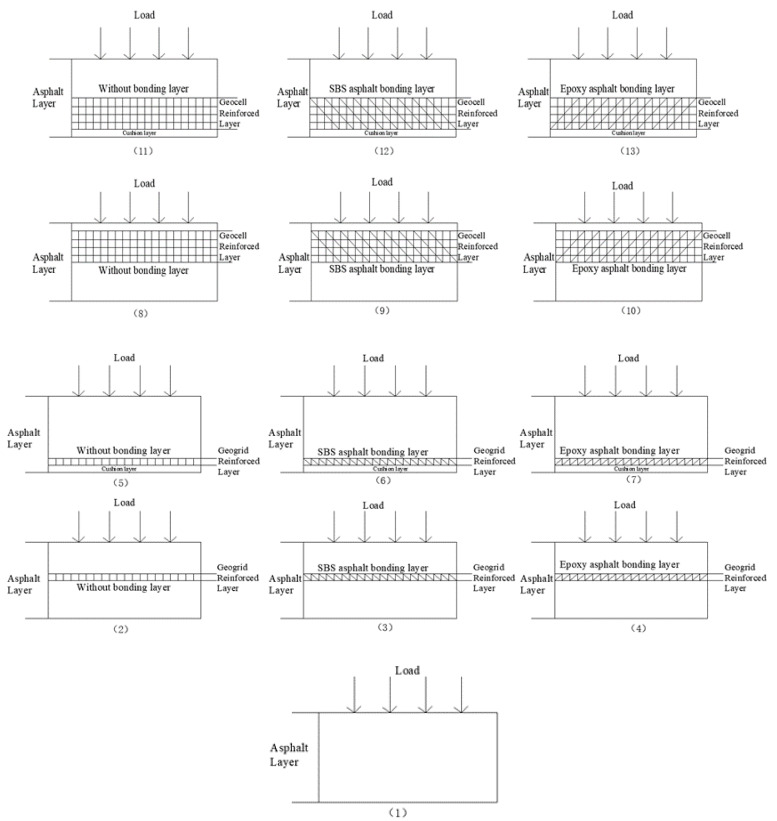
Reinforcement scheme of geosynthetics.

According to the types of geosynthetics, bonding layers and reinforced position, 12 different reinforced schemes are finally designed as shown in Figure 2. As can be seen, (1) is the unreinforced asphalt layer; (2) is the middle layer reinforced with geogrid, without bonding treatment; (3) is the middle layer reinforced with geogrid, bonded with SBS modified asphalt; (4) is the middle layer reinforced with geogrid, bonded with epoxy resin modified asphalt; (5) is the lower layer reinforced with geogrid, without bonding treatment; (6) is the lower layer reinforced with geogrid, bonded with SBS modified asphalt; (7) is the lower layer reinforced with geogrid, bonded with epoxy resin modified asphalt; (8) is the middle layer reinforced with geocell, without bonding treatment; (9) is the middle layer reinforced with geocell, bonded with SBS modified asphalt; (10) is the middle layer reinforced with geocell, bonded with epoxy resin modified asphalt; (11) is the lower layer reinforced with geocell, without bonding treatment; (12) is the lower layer reinforced with geocell, bonded with SBS modified asphalt; (13) is the lower layer reinforced with geocell, bonded with epoxy resin modified asphalt. The cushion layer is a thin protective layer to avoid direct contact between the bottom of the geosynthetic material and the top of the base layer.

Comparing (1) with the other 12 types of reinforced structures, we can see the effect of reinforcement under each of the above-mentioned reinforcement conditions. By comparing (2), (3), (4), (5), (6), (7) and (8), (9), (10), (11), (12), (13), the difference between geogrids and geocells under different reinforcement conditions can be obtained. By comparing (5), (6), (7), (11), (12), (13) and (2), (3), (4), (8), (9), (10), we can get the influence of the position of reinforcement under different reinforcement conditions. By comparing (2), (5), (8), (11), and (3), (6), (9), (12), and (4), (7), (10), (13), we can know the role of the bonding layer in the process of reinforcement under different reinforcement conditions.

### 3.2. Specimen Preparation

**Figure 3:**
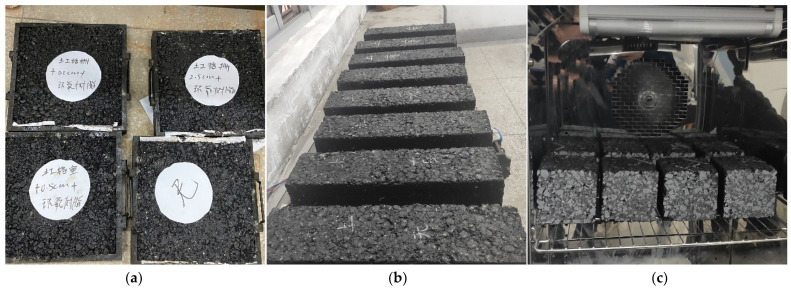
Geosynthetic-reinforced asphalt mixture specimens: (**a**) specimens of rut board; (**b**) beam specimens; (**c**) cube specimen.

At present, there is no standard specimen of geosynthetic-reinforced asphalt mixture, so this study refers to the standard specimen of asphalt mixture. The specimens of rut board were formed by wheel grinding and its size was 0.3 m × 0.3 m × 0.05 m, as shown in Figure 3a. The bending test in the asphalt mixture specification generally uses small beam specimens, but it is not suitable for adding geosynthetics. Therefore, to meet the requirements of reinforcement, a cuboid beam specimen with a size of 0.4 m × 0.4 m × 0.1 m was formed by the static pressing with a special mold, as shown in Figure 3b. The cube specimen is cut from the cuboid beam specimen. Although the exact position of the geosynthetic inside the specimen cannot be accurately determined during the cutting process, it can be guaranteed that each cube specimen can contain at least 3 complete cell units or 4 complete grid units; the size of the cube specimen is 0.1 m × 0.1 m × 0.1 m, as shown in Figure 3c.

### 3.3. Experimental Program and Test Equipment

In this study, the comparison between the results obtained from mechanical tests performed on geosynthetic-reinforced and unreinforced specimens was carried out to evaluate the performance of different reinforced schemes. The placement of geosynthetics in the asphalt layer mainly aims at three pavement distresses, which are cumulative permanent deformation of the pavement (rutting), bottom-up reflective cracks, and fatigue cracks under cyclic loading. Therefore, the above-mentioned 12 reinforced schemes were used to conduct rutting test, fatigue test, and bending creep test, and the whole experimental program is summarized in Table 4.

**Figure 4:**
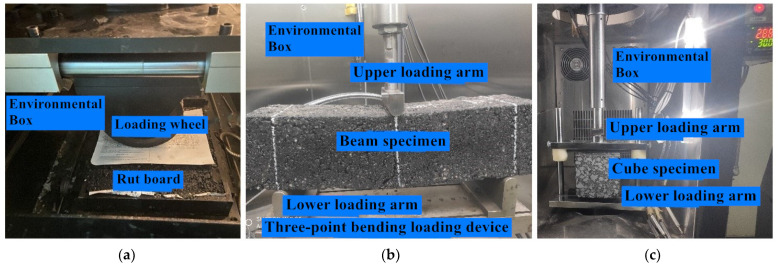
Test loading device: (**a**) rutting tests; (**b**) bending creep tests; (**c**) split fatigue tests.

Before the rutting test, the specimens were placed in the environmental chamber and cured for 6 h at the target temperature. The wheel pressure during rutting test was 0.7 MPa, which was the same as that under standard axle load. The loading device for rutting tests is shown in Figure 4a.

According to the research results [26,27], the bottom-up propagation process of reflected cracks in asphalt pavement is similar to the bottom-up propagation process of the cracks in asphalt mixture beam under bending. Therefore, three-point bending tests were carried out to test the crack resistance of the specimens and evaluate its anti-reflection crack performance. The loading device for bending tests is shown in Figure 4b.

In the split fatigue tests, the fixture strip of the standard specimen is arc-shaped. In order to prevent stress concentration when the two ends of the strip are in contact with the surface of cube specimen, a self-made long strip with a flat surface is used. The fatigue load is a stress-controlled haversine and no intermittent time is set to accelerate the fatigue process. Before fatigue tests, the specimens were also cured at the target temperature for 6 h, and the loading device is shown in Figure 4c.

In the above-mentioned tests, discard the difference according to the degree of dispersion of the test results, and supplement the test to ensure that the effective test is not less than three times.

## 4. Test Results

### 4.1. Rutting Test Results

**Figure 5:**
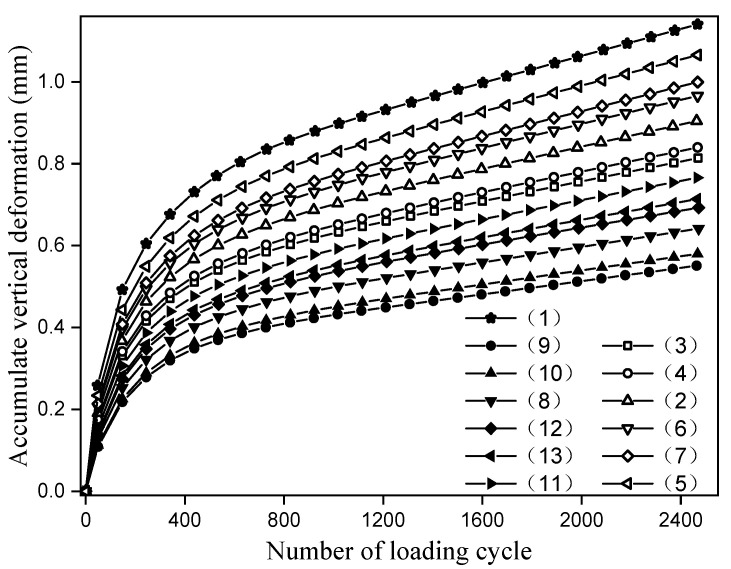
Rutting test results.

The vertical permanent deformation accumulated in asphalt pavement is the main cause of rutting. Rutting will seriously affect the comfort and safety of pavement and shorten its service life. The relationship between the number of loading cycle and the accumulate vertical deformation under the scheme in rutting test is shown in Figure 5. The vertical deformation of reinforced specimens is reduced to different degrees compared with the unreinforced specimens. By comparing the schemes (2) and (8), (3) and (9), (4) and (10), (5) and (11), (6) and (12) and (7) and (13), the vertical deformation of geocell-reinforced is smaller than that of geogrid-reinforced. According to the schemes (5) and (2), (6) and (3), (7) and (4), (11) and (8), (12) and (9) and (13) and (10), the vertical deformation of the middle layer reinforcement is smaller than that of the lower layer. Moreover, from the schemes (2), (5), (8), (11), and (3), (6), (9), (12), and (4), (7), (10), (13), the vertical deformation of the bonded layer is less than that of the unbonded layer, and the vertical permanent deformation of the bonded layer with SBS modified asphalt is smaller than that of epoxy modified asphalt.

The dynamic stability (*DS*) under different reinforcement schemes was calculated, and the calculation method is shown in Equation (1).

*DS* = [(*t*_2_ − *t*_1_) × *N*]/((*d*_2_ − *d*_1_) (1)

In the formula, *N* is the round-trip rolling speed of the test wheel. As can be seen from Table 4, the round-trip speed is 42 times/min; *d*_1_ and *d*_2_ are the rutting deformation of *t*_1_ and *t*_2_, respectively; *t*_1_ and *t*_2_ are 45 and 60 min. According to the rolling speed, the corresponding rolling cycles can be calculated as 1890 and 2520, respectively. Therefore, according to Figure 5 the deformation value of each reinforcement scheme under the above-mentioned wheel rolling cycle can be read. The dynamic stability calculated by Equation (1) is shown in Table 5 and abbreviated as *DS*.

### 4.2. Bending Creep Test Results

**Figure 6:**
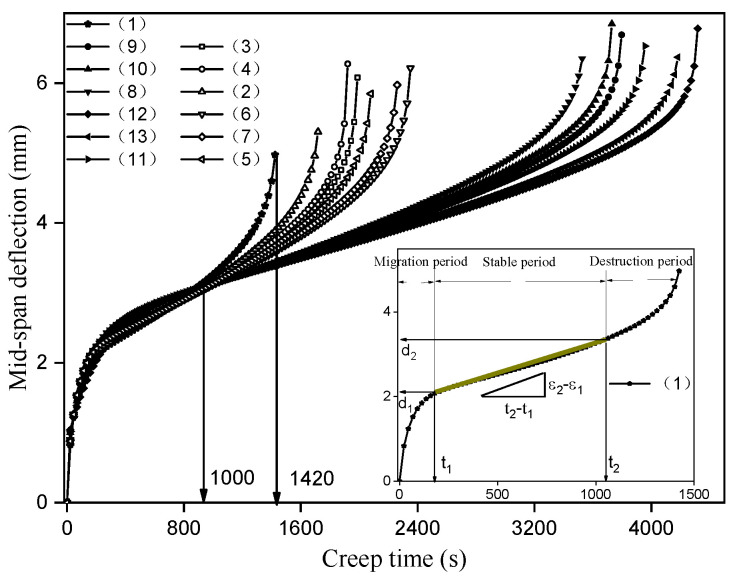
Bending creep test results.

Due to the stress concentration in the contact position between the bottom of asphalt layer and the crack under the action of vehicle, the crack spreads to the bottom of asphalt layer and expands from bottom to top, and finally forms the reflection crack. According to the experimental program in Table 3, the creep bending test is used to detect the crack resistance of the reinforcement scheme to evaluate its anti-reflective cracking performance. Ten percent of the bending failure load is taken as the creep load, and the bending failure load of unreinforced specimen at 15 °C is 21 kN, so the creep load is 2.1 kN. Under the action of creep load, the relationship curve between the mid-span deflection and the creep time is shown in Figure 6.

The variation trend of mid-span deflection with creep time is basically the same, which can be divided into migration period, stable period and destruction period. In addition, the duration of each reinforcement scheme in the above period is obviously different. In order to accurately evaluate the creep flexural resistance of the above reinforcement scheme, the creep bending rate is calculated, and the method is shown in Figure 6. After the bending creep curve enters the stable period, the mid-span deflection corresponding to the creep time at the start and end of the stage is read. Then, the bending strain at the bottom of the beam is calculated by the mid-span deflection, and the bending creep rate of the reinforcement scheme can be obtained.

Since the size of the specimen used is a prismatic beam with a length of 400 mm, a width of 100 mm, a height of 100 mm, and a span of 320 mm, the weight of the beam needs to be considered. The calculation process is shown in Equation (2).

(2) $$\left\{ \begin{array}{l} \sigma_{0}=\frac{3\times \left(2\times L\times F_{0}+q\times L^{2}-4\times q\times L_{1}^{2}\right)}{4\times b\times h^{2}}\times 10^{-6}\\ \varepsilon_{i}=\frac{24\times h\times \left(2\times L\times F_{0}+q\times L^{2}-4\times q\times L_{1}^{2}\right)}{\left(8\times L^{3}\times F_{0}+5\times q\times L^{4}-24\times q\times L^{2}\times L_{1}^{2}\right)}\times d_{i}=\alpha \times d_{i}\\ \varepsilon_{s}=\frac{\varepsilon_{2}-\varepsilon_{1}}{\left(t_{2}-t_{1}\right)/\sigma_{0}} \end{array}\right.$$

In the above formula, *σ*_0_ is the creep bending stress, *ε_i_* is the bending strain at the bottom of the beam, *b* and *h* are the width and height across the interrupt plane, respectively, L is the span, L_1_ is the distance from the end to the fulcrum, q is the mass per unit length, calculated by the following formula: q = (D − 1) × *b* × *h* × 1000 × 9.81, D is the density of asphalt mixture, F_0_ is the creep load, *d_i_* is the mid-span deflection, and *ε_s_* is the bending creep rate (1/(s·MPa)).

According to the physical and geometric characteristics of the beam specimens, the values of the various parameters in the above formula as well as the calculation results of the flexural stress *σ*_0_ and the constant coefficient *α* are shown in Table 6.

The above method was used to read the starting and ending positions of each reinforcement scheme in the stable period, and the bending creep rate was calculated according to Equation (2). The results are shown in Table 7.

From *t*_1_ and *t*_2_ in Table 7, it can be seen that the creep time of different reinforcement schemes in the migration period and the destruction period is not much different, and the main difference is in the stable period, that is to say, the reinforcement mainly acts on the stable period of the bending creep. The reinforced layer can absorb the stress at the crack tip and restrain the crack growth rate during the stable period, thus reducing the bending creep rate. The smaller the creep rate, the stronger the inhibition effect of the reinforced layer, and the better the crack resistance of the corresponding reinforcement scheme.

### 4.3. Split Fatigue Test Results

**Figure 7:**
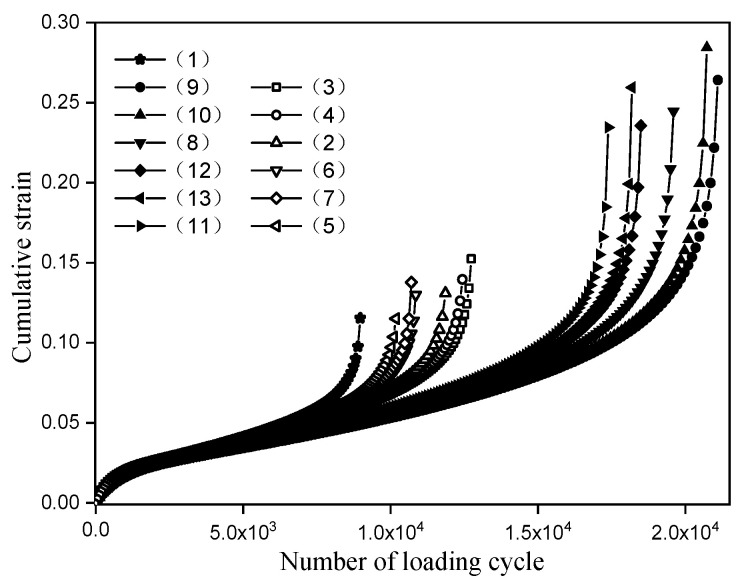
Split fatigue test results.

Fatigue crack is one of the main failure forms of asphalt pavement, and it is the process of initiation, propagation, and penetration of micro-cracks in asphalt mixture. The frequency and amplitude of the loading curve need to be determined before conducting the split fatigue test. According to the experimental program, the frequency is 10 Hz, and the loading amplitude is 30% of the splitting strength of the unreinforced specimens. The ultimate splitting load is 24 kN, so the loading amplitude is 7.2 kN. The cumulative strain of the split fatigue test under the above reinforcement scheme is shown in Figure 7.

**Figure 8:**
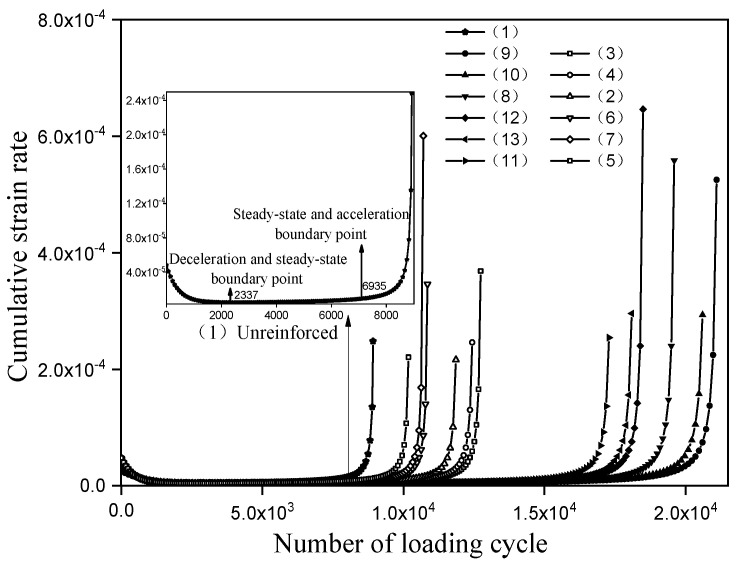
Cumulative strain rate under different reinforcement schemes.

As can be seen from Figure 7, the variation trend of the cumulative strain curve in the split fatigue test of each reinforcement scheme is basically the same. Under cyclic loading, the increasing rate of accumulated strain decreases gradually at first, then tends to be stable, and finally increases rapidly. In order to accurately describe the variation of accumulated strain of different reinforcement schemes at each stage of fatigue, the cumulative strain rate is calculated, as shown in Figure 8.

As can be seen from Figure 8, the variation trend of the cumulative strain rate curve under each reinforcement scheme is basically the same, with three obviously different variation stages: the initial rate decreases steadily, the middle rate increases slowly, and the late rate accelerates. They are called the deceleration fatigue stage, the steady-state fatigue stage, and the accelerated fatigue stage, respectively. Taking the unreinforced as an example, the position of the boundary between the deceleration and steady-state phase and the boundary between the steady-state and acceleration phase is shown in Figure 6. The corresponding number of loading cycle are 2337 and 6935, respectively, and the fatigue life at failure is 8894 cycles. According to the cumulative strain rate, it can also be known that the number of loading cycle in each fatigue stage and fatigue life is shown in Table 8.

## 5. Discussion

The data monitored during the rutting test is the cumulative vertical permanent deformation under different cycles, the data monitored in the bending creep test is the vertical displacement of the beam center with time under constant load, and the monitoring data in the splitting fatigue test is the accumulated strain under cyclic loading. Taking the unreinforced as a benchmark, the calculation method of the percentage change of each index after reinforcement is unified as shown in Equation (3).

(3) $$C_{I}=\left(I_{R}-I_{U}\right)/I_{U}$$

In the formula, *I_R_* is the index when reinforced, *I_U_* is the index when unreinforced, and *C_I_* is the percentage change of the index.

**Figure 9:**
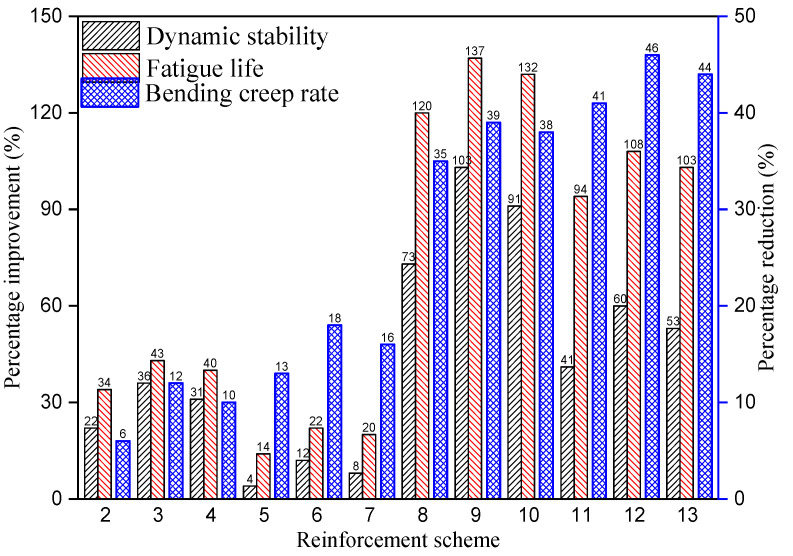
The variation of dynamic stability, fatigue life and bending creep rate under different reinforcement schemes.

According to Table 4, Table 6 and Table 7, using Equation (3) to calculate, we can get the percentage increase of dynamic stability and fatigue life, as well as the percentage decrease of bending creep rate, under the above 12 reinforcement schemes, as shown in Figure 9.

It can be seen from Figure 9 that the reinforcement scheme (9) has the largest increase in dynamic stability and fatigue life, which reached 103 and 137%, respectively. The largest reduction in bending creep rate is the reinforcement scheme (12), with a reduction of 46%. The results show that the reinforcement scheme (9) has the best anti-rutting and fatigue resistance performance, while the reinforcement scheme (12) has the best anti-reflective cracking performance. However, the improvement in dynamic stability and fatigue life of scheme (9) is 43 and 29% larger than scheme (12), and the reduction in bending creep rate of scheme (12) is only 7% larger than scheme (9).

## 6. Conclusions

The results obtained during this experimental investigation, carried out in order to study the performance of geosynthetics for asphalt pavement applications, allow several conclusive remarks to be made concerning rutting tests, bending creep tests and split fatigue tests.

(1) Geosynthetic reinforcement can restrain the vertical permanent deformation of asphalt mixture under the circulation wheel in rutting tests and improve the dynamic stability, extend the bending creep process of asphalt mixture under the constant load in bending creep tests, reduce the bending creep rate, decrease the cumulative strain of asphalt mixture under the cyclic load in fatigue tests, and improve the fatigue life.
(2) From the perspective of the types of geosynthetics, the reinforcement effect of geocells is much higher than that of geogrids. From the perspective of bonding layers, the reinforcement effect of SBS modified asphalt bonding layer is slightly better than that of epoxy modified asphalt bonding layer and both are better than that of no bonding layer. From the perspective of reinforced position, the rutting and fatigue resistance of middle reinforcement are better than that of the lower reinforcement, and the crack resistance of lower reinforcement is better than that of the middle reinforcement.
(3) According to the dynamic stability, bending creep rate and fatigue life, it can be seen that the reinforcement scheme (9) has the largest increase in dynamic stability and fatigue life, which are 103 and 137%, respectively. The reinforcement scheme (12) has the largest reduction in bending creep rate, reaching 46%. The improvement of dynamic stability and fatigue life of scheme (9) is 43 and 29% higher than that of scheme (12), while the reduction of bending creep rate of scheme (12) is only 7% higher than that of scheme (9).

## Figures and Tables

**Table 1:** Gradation of AC-13 asphalt mixture.

The Mass Percentage Passing										
Aggregate Sizes (mm)	16	13.2	9.5	4.75	2.36	1.18	0.6	0.3	0.15	0.075
Mass Percentage (%)	100	96.3	77.3	54.1	37.9	27.6	19.1	14.7	10.4	6.2

**Table 2:** Detection results of main technical indexes of grid coating.

Test Items	Test Results		Requirements
	SBS Asphalt	Epoxy Asphalt	
Penetration (25 °C, 100 g, 5 s, 0.1 mm)	57	52	40–60
Ductility (5 °C, 5 cm/min)	30	24	>20
Softening Point (°C)	81	77	>75
Elastic recovery (25 °C, %)	93	76	>75
Rotational viscosity (135 °C, Pa·s)	2.2	1.5	<3

**Table 3:** Performance of PP geocell and GF geogrid.

Polymer Type	Geosynthetics		Breaking Strength (kN/cm)	Elongation at Break (%)	Grid Size (cm × cm)	Thickness (mm)	Melting Point (°C)
Glass fiber	Geogrid	Bands	>1.2	<4	2.5 × 2.5	1.5	>1000
Polypropylene	Geocell		>0.275	<10	3 × 3	2	>160
		Node	>0.1	<10			

**Table 4:** Experimental program.

Tests	Specimen Type	Test Conditions
Rutting	Rut board	Temperature = 60 °C, Wheel pressure = 0.7 MPa, Round-trip rolling speed = 42 times/min.
Bending creep	Cuboid beam	Temperature = 15 °C, Creep load is taken as 10% of the bending strength.
Split fatigue	Cube	Temperature = 15 °C, Frequency = 10 Hz, Amplitude load is taken as 30% of the splitting strength.

**Table 5:** Rutting depth and dynamic stability under different reinforcement schemes.

Reinforcement Scheme			Dynamic Stability (Times/mm)
Unreinforced		(1) Unreinforced	6070
Geogrid	Middle layer	(2) Without bonding treatment	7407
		(3) SBS modified asphalt bonded	8264
		(4) Epoxy resin modified asphalt bonded	7937
	Lower layer	(5) Without bonding treatment	6289
		(6) SBS modified asphalt bonded	6803
		(7) Epoxy resin modified asphalt bonded	6579
Geocell	Middle layer	(8) Without bonding treatment	10,518
		(9) SBS modified asphalt bonded	12,336
		(10) Epoxy resin modified asphalt bonded	11,618
	Lower layer	(11) Without bonding treatment	8547
		(12) SBS modified asphalt bonded	9709
		(13) Epoxy resin modified asphalt bonded	9259

**Table 6:** Bending creep parameters of asphalt mixture beam specimens.

L (m)	F_0_ (N)	q (N/m)	L_1_ (m)	D (t/m^3^)	*h*	*b*	*σ*_0_ (MPa)	*α* (m^−1^)
0.32	2100	144.2	0.04	2.47	0.1m		1.018	5.845

**Table 7:** Rutting depth and dynamic stability under different reinforcement schemes.

Reinforcement Scheme			*t*_1_ (s)	*ε*_1_	*t*_2_ (s)	*ε*_2_	*ε_s_* (1/(s·MPa))
Unreinforced		(1) Unreinforced	226	0.01265	1085	0.02001	8.75 × 10^−6^
Geogrid	Middle layer	(2) Without bonding treatment	237	0.01299	1379	0.02218	8.21 × 10^−6^
		(3) SBS modified asphalt bonded	305	0.01376	1627	0.02382	7.74 × 10^−6^
		(4) Epoxy resin modified asphalt bonded	288	0.01358	1559	0.02338	7.85 × 10^−6^
	Lower layer	(5) Without bonding treatment	316	0.01393	1740	0.02456	7.62 × 10^−6^
		(6) SBS modified asphalt bonded	367	0.01448	2001	0.02605	7.14 × 10^−6^
		(7) Epoxy resin modified asphalt bonded	339	0.01424	1921	0.0256	7.32 × 10^−6^
Geocell	Middle layer	(8) Without bonding treatment	294	0.01446	3277	0.03105	5.65 × 10^−6^
		(9) SBS modified asphalt bonded	328	0.01474	3548	0.03141	5.3 × 10^−6^
		(10) Epoxy resin modified asphalt bonded	316	0.01465	3480	0.03156	5.41 × 10^−6^
	Lower layer	(11) Without bonding treatment	345	0.01481	3729	0.03191	5.21 × 10^−6^
		(12) SBS modified asphalt bonded	384	0.01474	4133	0.03219	4.76 × 10^−6^
		(13) Epoxy resin modified asphalt bonded	362	0.01472	3978	0.03212	4.88 × 10^−6^

**Table 8:** The number of loading cycle in each fatigue stage and fatigue life.

Reinforcement Scheme			Deceleration	Steady-State	Acceleration	Fatigue Life
Unreinforced		(1) Unreinforced	2337	4598	1959	8894
Geogrid	Middle layer	(2) Without bonding treatment	2503	7537	1789	11,879
		(3) SBS modified asphalt bonded	2714	8366	1659	12,739
		(4) Epoxy resin modified asphalt bonded	2638	8216	1583	12,437
	Lower layer	(5) Without bonding treatment	2261	6407	1508	10,176
		(6) SBS modified asphalt bonded	2412	6935	1507	10,854
		(7) Epoxy resin modified asphalt bonded	2337	6784	1583	10,704
Geocell	Middle layer	(8) Without bonding treatment	3618	13,668	2312	19,598
		(9) SBS modified asphalt bonded	4271	14,448	2387	21,106
		(10) Epoxy resin modified asphalt bonded	3894	14,196	2513	20,603
	Lower layer	(11) Without bonding treatment	3015	12,261	2010	17,286
		(12) SBS modified asphalt bonded	3317	12,964	2211	18,492
		(13) Epoxy resin modified asphalt bonded	3015	13,166	1909	18,090

## Data Availability

The data that support the finding of this study are available from the corresponding author upon reasonable request.

## References

[1] Han C., Ma T., Chen S. (2021). Asphalt pavement maintenance plans intelligent decision model based on reinforcement learning algorithm. Constr. Build. Mater.

[2] Ma F., Dong W., Fu Z., Wang R., Huang Y., Liu J. (2021). Life cycle assessment of greenhouse gas emissions from asphalt pavement maintenance: A case study in China. J. Clean. Prod..

[3] Pan Y., Han D., Yang T., Tang D., Huang Y., Tang N., Zhao Y. (2021). Field observations and laboratory evaluations of asphalt pavement maintenance using hot in-place recycling. Constr. Build. Mater.

[4] Solatiyan E., Bueche N., Carter A. (2020). A review on mechanical behavior and design considerations for reinforced-rehabilitated bituminous pavements. Constr. Build. Mater.

[5] Mirzapour M.S., Karim M.R., Khodaii A. (2014). Improving Rutting Resistance of Pavement Structures Using Geosynthetics: An Overview. Sci. World J..

[6] Torre I., Perez C.M.A., Zamanillo V.A., Fresno D.C. (2015). Experimental study of the behaviour of different geosynthetics as anti-reflective cracking systems using a combined-load fatigue test. Geotext. Geomembr..

[7] Ferrotti G., Canestrari F., Virgili A. (2011). A strategic laboratory approach for the performance investigation of geogrids in flexible pavements. Constr. Build. Mater.

[8] Ferrotti G., Canestrari F., Pasquini E. (2012). Experimental evaluation of the influence of surface coating on fiberglass geogrid performance in asphalt pavements. Geotext. Geomembr..

[9] Pasquini E., Bocci M., Ferrotti G. (2013). Laboratory characterization and field validation of geogrid-reinforced asphalt pavements. Constr. Build. Mater.

[10] Nithin S., Rajagopal K., Veeraragavan A. (2015). State-of-the Art Summary of Geosynthetic Interlayer Systems for Retarding the Reflective Cracking. Indian Geotech. J..

[11] Dehghan Z., Modarres A. (2017). Evaluating the fatigue properties of hot mix asphalt reinforced by recycled PET fibers using 4-point bending test. Constr. Build. Mater.

[12] Subaida E.A., Chandrakaran S., Sankar N. (2009). Laboratory performance of unpaved roads reinforced with woven coir geotextiles. Geotext. Geomembr..

[13] Bertuliene L., Oginskas R., Bulevicius M. (2011). Research of Rut Depth in Asphalt Pavements Reinforced with Geosynthetic Materials. Proc. Int. Conf. Environ. Eng..

[14] Correia N.S., Zornberg J.G. (2015). Mechanical Response of Flexible Pavements Enhanced with Geogrid-reinforced Asphalt Overlays. Geosynth. Int..

[15] Qadir A., Gazder U., Choudhary K. (2021). Statistical analysis for comparing and predicting rutting resistance of asphalt pavements with rigid and flexible geogrid layers. Constr. Build. Mater..

[16] Khodaii A. (2009). Effects of Geosynthetics on Reduction of Reflection Cracking in Asphalt Overlays. Geotext. Geomembr..

[17] Nejad F.M., Asadi S., Fallah S. (2016). Statistical-experimental Study of Geosynthetics Performance on Reflection Cracking Phenomenon. Geotext. Geomembr..

[18] Ling J., Wei F., Gao J. (2019). New Test Method for Measuring Reflective Cracking in Hot-Mix Asphalt Overlay Pavements. Transp. Res. Rec. J. Transp. Res. Board.

[19] Lv D.W. (2014). Research on the Comprehensive Technology of Cement Concrete Pavement with Asphalt Overlay in Expressway. Ph.D. Thesis.

[20] Arsenie I.M., Chazallon C., Duchez J.L. (2017). Laboratory Characterisation of the Fatigue Behaviour of a Glass Fibre Grid-reinforced Asphalt Concrete Using 4PB Tests. Road Mater. Pavement.

[21] Kumar V.V., Saride S. (2017). Use of Digital Image Correlation for the Evaluation of Flexural Fatigue Behavior of Asphalt Beams with Geosynthetic Interlayers. Transp. Res. Rec. J. Transp. Res. Board.

[22] Button J.W., Lytton R.L. (2007). Guidelines for Using Geosynthetics with Hot-Mix Asphalt Overlays to Reduce Reflective Cracking. Transp. Res. Rec..

[23] Taherkhani H., Jalali M. (2016). Investigating the Performance of Geosynthetic-reinforced Asphaltic Pavement Under Various Axle Loads Using Finite-element method. Road Mater. Pavement.

[24] Wang X.R., Zhang X.D., Zhu Y.S. (2020). Fatigue damage characteristics of geocell-reinforced asphalt mixture. Constr. Build. Mater.

[25] Solatiyan E., Bueche N., Vaillancourt M. (2020). Permeability and Mechanical Property Measurements of Reinforced Asphalt Overlay with Paving Fabrics Using Novel Approaches. Mater. Struct..

[26] Saride S., Kumar V.V. (2017). Influence of Geosynthetic-interlayers on the Performance of Asphalt Overlays on Pre-cracked Pavements. Geotext. Geomembr..

[27] Safavizadeh S.A., Wargo A., Guddati M., Kim Y. (2015). Investigating Reflective Cracking Mechanisms in Grid-reinforced Asphalt Specimens: Use of Four-point Bending Notched Beam Fatigue Tests and Digital Image Correlation. Transp. Res. Rec..

